# Mesenchymal stem cell-conditioned medium accelerates type 2 diabetic wound healing by targeting TNF and chemokine signaling

**DOI:** 10.3389/fcell.2025.1659444

**Published:** 2025-09-24

**Authors:** Long Huang, Zhongbao Lin, Haiyun Liu, Xiankun Lin, Naishun Liao, Xiaodan Wu

**Affiliations:** ^1^ Department of Respiratory and Critical Care Medicine, Fuzhou University Affiliated Provincial Hospital, Fujian Provincial Hospital, Shengli Clinical Medical College of Fujian Medical University, Fuzhou, China; ^2^ Department of Critical Care Medicine, Fuzhou University Affiliated Provincial Hospital, Fujian Provincial Hospital, Shengli Clinical Medical College of Fujian Medical University, Fuzhou, China; ^3^ Shengli Clinical Medical College of Fujian Medical University; Department of Emergency, Fujian Provincial Hospital; Fuzhou University Affiliated Provincial Hospital; Fujian Provincial Key Laboratory of Emergency Medicine, Fuzhou, China; ^4^ The United Innovation of Mengchao Hepatobiliary Technology Key Laboratory of Fujian Province, Mengchao Hepatobiliary Hospital of Fujian Medical University, Fuzhou, China; ^5^ Department of Anesthesiology, Shengli Clinical Medical College of Fujian Fuzhou University Affiliated Provincial Hospital, Fuzhou, China

**Keywords:** adipose tissue-derived mesenchymal stem cells, conditioned medium, type 2 diabetes, skin wound, regeneration

## Abstract

**Introduction:**

Given the crucial role of paracrine signaling in the therapeutic function of adipose tissue-derived mesenchymal stem cells (ADSCs) for skin wound repair, this study aimed to evaluate the efficacy of ADSC-conditioned medium (ACM) in enhancing type 2 diabetic (T2D) wound healing.

**Methods:**

The effect of ACM on the viability and angiogenesis of human umbilical vein endothelial cells (HUVECs) was first evaluated using the CCK-8 assay and q-PCR analysis, respectively. Next, a T2D rat model was established through the combination of a high-fat diet and streptozotocin (STZ). Following the establishment of full-thickness skin defects in T2D rats, ACM or serum-free cultured medium was daily injected around the wound edges for 7 days. Afterward, the skin wound healing rate was analyzed, and the skin tissues were assessed by histopathological examination. The mRNA levels of TNF-α, IL-1β, IL-6, COX-2, IL-12, and IFN-γ were evaluated by q-PCR analysis. Additionally, transcriptome sequencing and immunohistochemistry were performed to reveal the potential mechanisms of ACM in T2D skin wound healing.

**Results:**

ACM significantly enhanced HUVEC proliferation and angiogenesis while upregulating the expression of EGF, bFGF, VEGF, and KDR. In T2D rats, ACM accelerated wound closure and suppressed pro-inflammatory mediators (TNF-α, IL-1β, IL-6, COX-2, IL-12, and IFN-γ). Notably, transcriptome analysis revealed ACM-mediated downregulation of TNF and chemokine signaling pathways.

**Discussion:**

ACM promotes diabetic wound healing through dual mechanisms: (1) stimulating vascularization by inducing growth factor expression and (2) modulating the inflammatory microenvironment by inhibiting TNF/chemokine cascades. These findings position ACM as a promising cell-free therapy for impaired wound healing in diabetes.

## 1 Introduction

Skin wound healing is a complex process involving multiple stages, such as hemostasis, inflammation, angiogenesis, and remodeling, which requires the coordinated effort of various cell types and signaling pathways (Freedman et al., 2023; Martin and Nunan, 2015). There are several factors, such as ischemia, diabetes, age, nutrition, hormones, obesity, infection, smoking, alcoholism, and radiation and chemotherapy, which can influence one or more stages of this process, resulting in improper or impaired wound healing (Roux et al., 2025). In particular, delayed wound healing in diabetic patients is increasing globally due to the lack of effective intervention strategies and the widespread prevalence of diabetes (De Souza et al., 2023).

Given their excellent immunoregulation, multidirectional differentiation ability, and paracrine function, adipose-derived mesenchymal stem cells (ADSCs) have emerged as a novel and promising strategy for treating diabetic wounds in both preclinical and clinical studies (Yan et al., 2024; Carstens et al., 2021; Huerta et al., 2023). However, the effectiveness of ADSCs in repairing diabetic wounds is limited by their low engraftment efficiency, which could be partially attributed to the stark contrast between optimized *in vitro* culture conditions and the harsh pathological microenvironment of chronic wound sites (Yu et al., 2023). Therefore, further research is needed to improve the efficacy of ADSC therapy for diabetic wound healing. Recently, increasing evidence has suggested that the paracrine function of ADSCs plays a leading role in skin wound regeneration (Ma et al., 2023; Ren et al., 2024; Wei et al., 2024; Wang et al., 2024). In particular, instead of mesenchymal stem cells (MSCs), using MSC-conditioned medium or secretome also provides a therapeutic potential for reducing irradiated skin injuries (Lin et al., 2023) and scar fibrosis (Zhang et al., 2021; Wang et al., 2024). More importantly, this cell-free strategy effectively avoids the potential limitation of low cell engraftment of MSCs for wound healing.

In this study, we investigated whether ADSC-conditioned medium (ACM) can be used for accelerating diabetic wound healing in rats. To achieve this purpose, we evaluated the therapeutic effect of ACM on skin wounds both *in vitro* and *in vivo* and the potential mechanism of ACM in diabetic wound healing. The results suggest that ACM may offer a promising strategy to promote diabetic wound recovery.

## 2 Materials and methods

### 2.1 Animals

Twenty adult male Sprague–Dawley (SD) rats (weighing 180 g–200 g) were obtained from the Shanghai Slack Laboratory Animal Center (license number: SCXK hu 2022-0004). All rats were housed in a standard specific pathogen-free (SPF) barrier environment at 20 °C–26 °C and 40%–70% humidity under a 12 h/12 h light–dark cycle. All animal experiments were approved by the Experimental Animal Ethics Center of Mengchao Hepatobiliary Hospital of Fujian Medical University (MCHH-AEC-2022-08). All experiments were designed and reported in accordance with the Animal Research: Reporting of *In Vivo* Experiments (ARRIVE) guidelines 2.0.

### 2.2 Preparation of ADSC-conditioned medium

The ADSCs were isolated and cultured according to previously published methods (Liao et al., 2017). In brief, adipose tissues were obtained from the inguinal region of male SD rats (n = 5) and washed with PBS solution. The tissues were then cut into small fragments and digested with 0.1% type I collagenase, followed by neutralization with α-MEM containing 10% FBS. Subsequently, the cells were cultured at a density of 1 × 10^6^ cells/mL in T-75 plates. ADSCs at passage 3 were collected and cultured at a density of 2 × 10^6^ cells per 10 cm plate. After overnight cell adhesion, the cultured ADSCs were washed with PBS solution to remove residual serum and then replaced with serum-free medium (YOCON, China) for 48 h. After incubation, the conditioned medium (10 mL/2 × 10^6^ ADSCs) was collected and centrifuged at 3,000 *g* for 5 min to remove any cell debris. Furthermore, the ADSC-conditioned medium was concentrated using ultrafiltration with a tangential flow filtration capsule (Pall, United States) containing a 3-kDa molecular weight cut-off membrane, following the manufacturer’s instructions. Finally, the concentration of the ADSC-conditioned medium was analyzed using a BCA assay kit (TransGen Biotech, China) and stored at −80 °C. A concentration of 100 ng/mL ADSC-conditioned medium was used in the present study.

### 2.3 HUVEC culture

The human umbilical vein endothelial cell (HUVEC) line was obtained from the National Institutes for Food and Drug Control (Beijing, China) and cultured with RPMI 1640 containing 10% FBS supplemented with 2% FBS, VEGF, IGF-1, and EGF at 37 °C/5% CO_2_. For the tubule formation assay, 96-well plates were pre-coated with Matrigel (Corning, 10 mg/mL, 1:50 dilution in EGM-2) for 1 h at 37 °C to simulate the basement membrane matrix, as standardized in angiogenesis assays.

### 2.4 Cell viability assay

HUVECs were cultured at a density of 1 × 10^4^ cells per well in 96-well plates. After overnight cell adhesion, the cell supernatants were removed and replaced with 100 μL ADSC-conditioned medium, while the cells treated with serum-free medium were used as the negative control. After incubation for 24 h or 48 h, cell viability was evaluated using a CCK-8 assay kit (TransGen Biotech, China), according to the manufacturer’s instructions.

### 2.5 Quantitative real-time PCR analysis

Total RNA was collected using a TRIzol reagent kit (TransGen Biotech, China) following the manufacturer’s instructions. Afterward, mRNA was reverse-transcribed into cDNA using a cDNA synthesis kit (Roche, Germany). The quantitative real-time PCR analysis was performed in an ABI StepOnePlus Real-time PCR System (Carlsbad, United States), and the PCR conditions were as follows: 95 °C for 15 s, 60 °C for 30 s, and 70 °C for 30 s, for a total of 40 cycles. The primer sequences are listed in Table 1. The 2^-△△Ct^ formula was used to analyze the relative gene expression.

**TABLE 1:** Primer sequences.

Gene	Forward primer	Reverse primer
TNF-α	CAGAGGGAAGAGTTCCCCAG	CCTTGGTCTGGTAGGAGACG
IL-1β	CACCTCTCAAGCAGAGCACAG	GGGTTCCATGGTGAAGTCAAC
IL-6	CACTGGTCTTTTGGAGTTTGAG	GGACTTTTGTACTCATCTGCAC
COX-2	CGGAGGAGAAGTGGGGTTTAGGAT	TGGGAGGCACTTGCGTTGATGG
IL-12	AGTTCTTCGTCCGCATCCAG	CTTGCACGCAGAT ATTCGCC
IFN-γ	CAACCCACAGATCCAGCACA	TCAGCACCGACTCCTTTTCC
VEGF	CCCAGAAGTTGGACGAAAA	TGAGTTGGGAGGAGGATG
EGF	ACACGGAGGGAGGCTACA	GTAGCCTCCCTCCGTGTT
bFGF	CGCACCCTATCCCTTCACA	CAACGACCAGCCTTCCAC
KDR	ACTCCTCCTCATTCAGCG	GGGTCCCACAACTTCTCA
β-actin	GTGGACA TCCGCAA AGAC	AAAGGGTGTAACGC AACTA

### 2.6 Diabetic skin-injured model and ACM treatment

The type 2 diabetic (T2D) model was established using a previously described method (Liao et al., 2017). In brief, the SD rats (n = 10) were fed with a high-fat diet (HFD) containing 66.5% normal chow, 20% sucrose, 10% lard, 2% cholesterol, and 1.5% cholate. After being fed with the HFD for 4 weeks, all rats were administered 25 mg/kg of streptozotocin (STZ) by intraperitoneal injection twice/week for 2 weeks. Rats treated with STZ and exhibiting a non-fasting blood glucose level ≥11.1 mmol/L were considered successful in establishing the T2D model. Next, the T2D rats were anesthetized with 40 mg/kg of pentobarbital sodium, and a full-thickness skin defect of 1 cm in diameter was created using a previously described method (Hur et al., 2017). Subsequently, the rats were randomly (random table method) divided into T2D skin-injured model and ACM groups (n = 5/group) and housed separately. Rats in the ACM group were treated daily with 100 μL ACM administered intradermally around the wound edges for 7 days, while model rats received an equal volume of serum-free medium. Normal rats (n = 5) were used as the negative control. On the 5th day after the last ACM treatment (the 12th day in total), all rats were euthanized with 100 mg/kg of pentobarbital sodium, and the wounds were harvested for further evaluation. All animals were selected at random for outcome assessment.

### 2.7 Histological examination

Tissues were collected and fixed in 4% paraformaldehyde for 24 h and then paraffin-embedded and sectioned into slices. Tissue sections were evaluated with hematoxylin and eosin (HE) staining and Masson staining, respectively. Finally, a double-blind histological examination was performed using an ortho-microscope (Zeiss, Germany) by two expert pathologists.

### 2.8 RNA sequencing

Total RNA from skin tissues was subjected to polyA-selected RNA-sequencing on the Illumina HiSeq X10 platform in a blinded manner. Using the DESeq2 package, RNA-seq analysis was carried out to determine the different gene expression (DEG) among three groups: normal vs. model and ACM vs. model. False discovery rate (FDR) of < 0.05 and fold change of ≥ 2 or ≤ 2 were the principles for DEG screening. Gene Ontology (GO) analysis was used to analyze the gene functions of the DEGs, and the Kyoto Encyclopedia of Genes and Genomes (KEGG) analysis was used to target the DEGs’ enrichment pathway.

### 2.9 Immunohistochemistry

The skin wound sections were drenched in a citrate antigen retrieval solution (Beyotime Institute of Biotechnology, China) and heat-treated in a pressure cooker for 2 min, naturally cooled to RT, and washed with PBS buffer three times. Following incubation with 3% H_2_O_2_ for 10 min, the sections were blocked with 5% BSA for 30 min. The sections were then incubated overnight at 4 °C with primary antibodies against TNF-α, NF-κB, p-NF-κB, MAPK, p-MAPK, CXCL1, CXCL2, and CXCL8 at a dilution ratio of 1:200 . After washing three times with PBS, the sections were incubated with the secondary antibody at RT for another 2 h, followed by staining with DAB. The samples were observed using an ortho-microscope (Zeiss, Germany) in a blinded manner by the assessors.

### 2.10 Statistical analysis

All quantitative data were expressed as the mean ± standard deviation. GraphPad Prism version 9.0 (GraphPad Software, United States) was used for statistical analysis. The ANOVA was used to evaluate the significant differences among three independent groups, while the two-tailed paired sample Student’s t-tests were used to evaluate the significant differences between two groups. *p* < 0.05 was considered a statistically significant difference.

## 3 Results

### 3.1 ACM promotes HUVEC angiogenesis and proliferation *in vitro*

**FIGURE 1:**
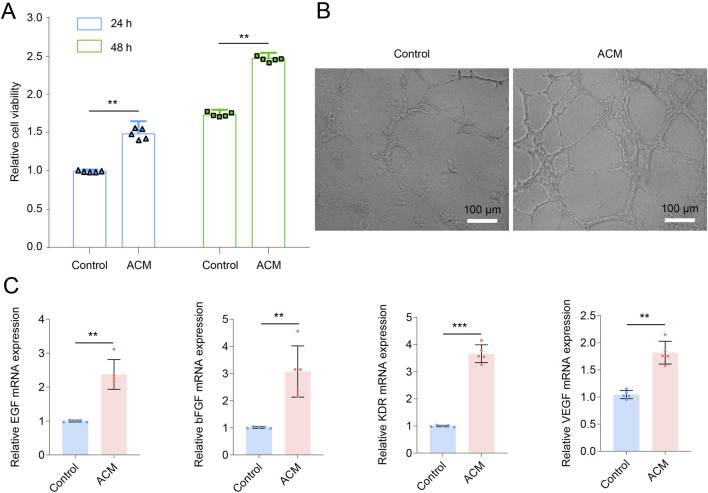
ACM promotes vascular cell proliferation and angiogenesis. **(A)** ACM promotes HUVEC proliferation. **(B)** Representative images of HUVECs after ACM treatment (scale bar, 100 μm). **(C)** Relative mRNA expression of EGF, bFGF, VEGF, and KDR in HUVECs after ACM treatment.

Flow cytometry analysis showed that ADSCs typically expressed CD73 and CD90 while lacking expression of CD45, CD19, and CD34 (Supplementary Figure S1), suggesting the successful isolation and culture of ADSCs in this study. The impaired skin wound healing in diabetic individuals is largely attributed to diabetic angiopathy, which is characterized by the dysfunction and impairment of the arteries throughout the body (Jin et al., 2022). We, therefore, investigated the effect of ACM on the angiogenesis and proliferation of vascular cells *in vitro*. After incubation with ACM for 24 or 48 h, the viability of HUVECs was significantly increased (Figure 1A), suggesting that ACM promoted HUVEC proliferation. After 2 days of continuous ACM incubation, HUVECs showed vascular-like morphological changes (Figure 1B), and the expression of genes associated with angiogenesis, including EGF, bFGF, VEGF, and KDR, was significantly upregulated after ACM treatment (Figure 1C), implying that ACM promotes HUVEC angiogenesis *in vitro*.

### 3.2 ACM accelerates T2D skin wound healing

**FIGURE 2:**
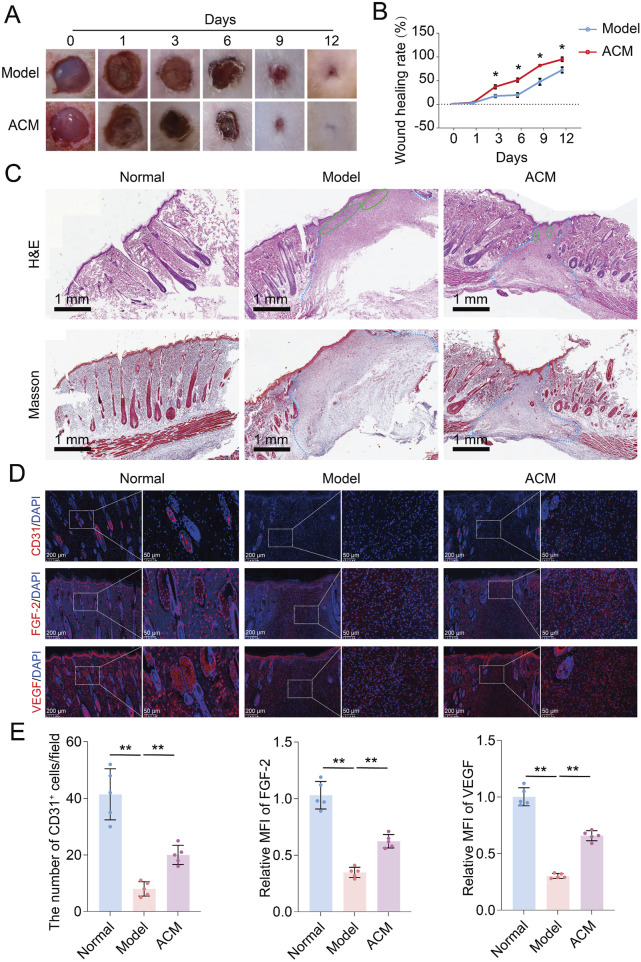
ACM accelerates T2D skin wound healing in rats. **(A)** General observation of skin wounds after ACM treatment. **(B)** Skin wound healing rate after ACM treatment. **(C)** Histopathological changes in skin wounds after ACM treatment by HE and Masson staining, respectively (scale bar, 1 mm). The area within the blue dotted line represents the damaged region. The green oval shape represents the area of inflammation. **(D)** Immunofluorescence expression of CD31, FGF-2, and VEGF in skin wounds after ACM treatment. Scale bar = 200 μm and 50 μm, respectively. **(E)** Number of CD31^+^ cells and the relative MFI of FGF-2 and VEGF expression in skin wounds after ACM treatment.

Based on the pro-angiogenic potential of ACM in vascular cells, we established a T2D skin wound model to further evaluate its therapeutic effects. As shown in Figures 2A, B, the skin wound healing rate of T2D rats was significantly improved by the continuous ACM treatment for 7 days compared to that of the model group. Moreover, increased tissue regeneration and decreased inflammatory infiltration were also observed in the ACM group compared to those in the model group (Figure 2C). Given the excellent performance of ACM on angiogenesis *in vitro*, we also investigated the beneficial effect of ACM on angiopathy *in vivo*. We found that the number of CD31^+^ cells and the expression of FGF-2 and VEGF were markedly increased in the ACM group compared to those in the model group (Figures 2D, E), suggesting that ACM could also improve angiopathy *in vivo*. Therefore, these data suggest that ACM accelerates T2D skin wound healing.

### 3.3 ACM inhibits T2D skin wound inflammation

**FIGURE 3:**
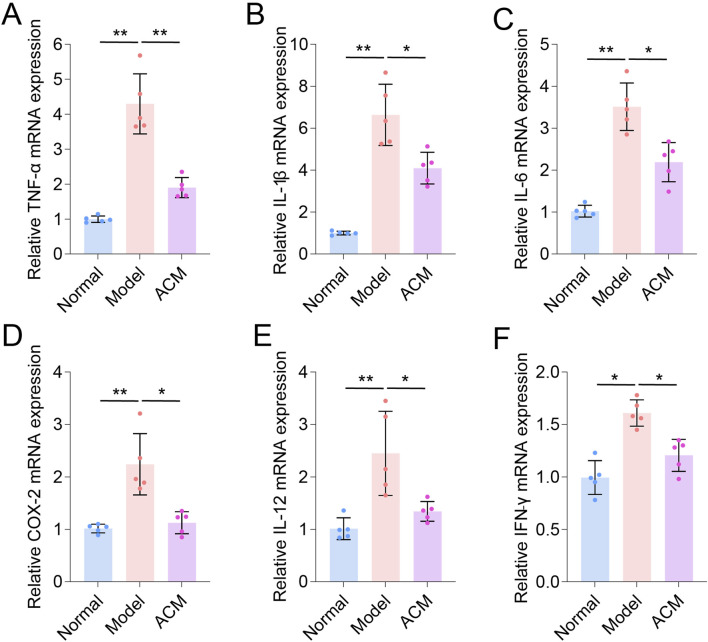
ACM inhibits the mRNA level of inflammatory factors in T2D skin wounds. The relative mRNA expression of TNF-α **(A)**, IL-1β **(B)**, IL-6 **(C)**, COX-2 **(D)**, IL-12 **(E)**, and IFN-γ **(F)** in skin wounds.

**FIGURE 4:**
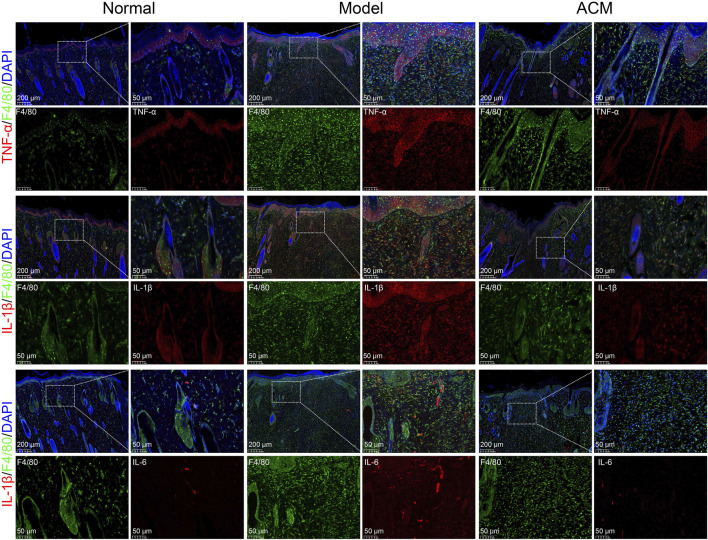
ACM inhibits the expression of inflammatory factors in macrophages. The immunofluorescence expression of TNF-α, IL-1β, and IL-6 in F4/80^+^ macrophages in skin wounds after ACM treatment. Scale bar = 200 μm and 50 μm, respectively.

Given that excessive inflammation is a typical characteristic of skin wounds (Huang et al., 2022), we further analyzed the inflammatory genes in T2D skin wound tissues. As shown in Figure 3, the mRNA expression levels of TNF-α, IL-1β, IL-6, COX-2, IL-12, and IFN-γ were significantly increased in T2D skin wounds compared to those in normal skin tissues, indicating excessive inflammation. However, these levels were effectively decreased after ACM treatment compared to those in the model groups, suggesting that ACM could inhibit this excessive inflammation. Moreover, we found that ACM treatment reduced inflammatory cell infiltration. This included a reduction in both the CD3^+^ T cells and F4/80^+^ macrophages (Supplementary Figure S3). Furthermore, the expression of TNF-α, IL-1β, and IL-6 was significantly decreased in the ACM-treated groups (Figure 4). Taken together, these data suggest that ACM mitigates the inflammatory response in skin wounds of diabetic rats.

### 3.4 ACM promotes T2D skin wound healing by targeting the TNF and chemokine signaling pathway

**FIGURE 5:**
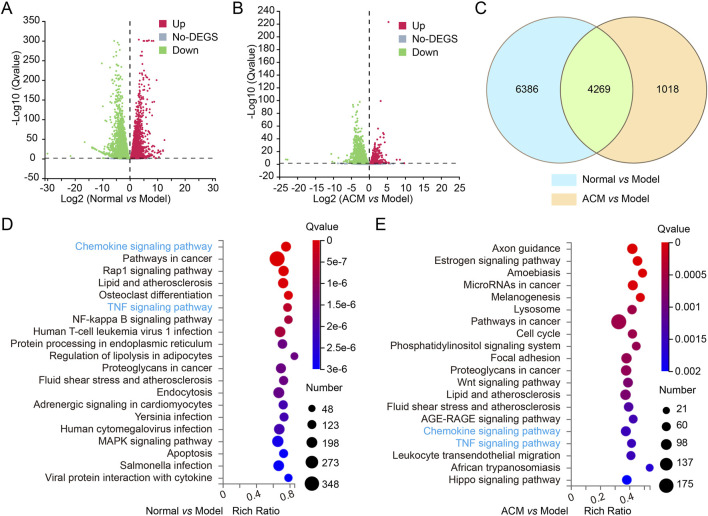
Transcriptome sequencing analysis of T2D skin wound tissues in rats. The volcano plot for differential gene expression of normal vs. model groups **(A)** and ADSC vs. model **(B)** groups. The gray pixel represents a gene where the difference in expression is not significant, while red and green pixels represent those that are significant. **(C)** Venn diagrams exhibiting the number of identified genes and the overlay of these identified genes. GO annotation and pathway enrichment analysis in normal vs. model groups **(D)** and ADSC vs. model **(E)** groups.

The potential molecular mechanism of ACM in accelerating T2D skin wound healing was further explored by RNA sequencing. Volcano plot analysis revealed differential expression of 10,655 genes was different between the normal and model groups (normal vs. model) and 5,287 genes between the ACM and model groups (ACM vs. model); a total of 4,269 genes were common to both comparisons (Figures 5A–C). GO annotation and pathway enrichment analysis showed that the upregulation of TNF and chemokine signaling was observed in the model group (compared with the normal group), while downregulation of TNF and chemokine signaling was clearly observed in the ACM group compared with the model groups (Figures 5D, E), suggesting that the potential molecular mechanism of ACM in T2D skin wound healing is by targeting the TNF and chemokine signaling pathway.

**FIGURE 6:**
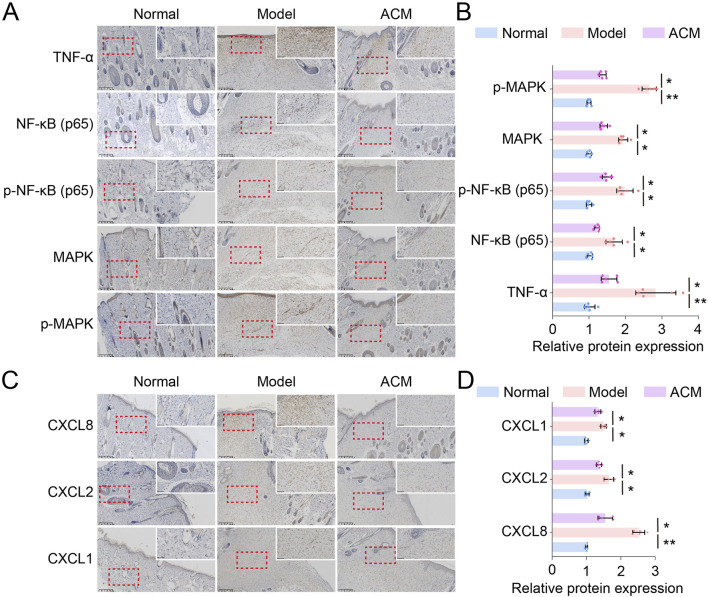
ACM downregulates TNF and chemokine signaling in T2D skin wound tissues. **(A)** Representative images of TNF-α, NF-κB, p-NF-κB, MAPK, and p-MAPK expression in skin tissues. Scale bars = 200 μm. **(B)** Relative expressions of TNF-α, NF-κB, p-NF-κB, MAPK, and p-MAP . **(C)** Representative images of CXCL1, CXCL2, and CXCL8 expression in skin tissues. Scale bars = 200 μm. **(D)** Relative expressions of CXCL1, CXCL2, and CXCL8.

To confirm the RNA sequencing results, we further evaluated the protein expression of the main regulators in TNF and chemokine signaling. As shown in Figure 6, the TNF signaling-related proteins, including TNF-α, NF-κB, p-NF-κB, MAPK, and p-MAPK, and the chemokine signaling-related proteins, including CXCL1, CXCL2, and CXCL8, were all downregulated by ACM treatment in T2D skin wounds, suggesting that ACM accelerated T2D skin wound healing via the downregulation of TNF and chemokine signaling.

## 4 Discussion

Angiogenesis is an essential part of skin wound regeneration, and it is also prone to being impaired by the diabetes status (Fan et al., 2024), excessive inflammation (Guo et al., 2023), oxidative stress, and other chronic wound conditions (De Wolde et al., 2021). Given the excellent performance of MSCs in promoting vasculogenesis through paracrine factors (e.g., VEGF, EGF, and bFGF) (Guillamat-Prats, 2021), MSC secretome or conditioned medium provides a new strategy for accelerating angiogenesis of skin wounds (Hade et al., 2022). Adipose tissue-derived ACM was assessed in this study to confirm its beneficial effects on angiogenesis, owing to the abundant availability and easy accessibility of adipose tissues (Bunnell, 2021). As expected, ACM effectively promoted HUVEC proliferation and angiogenesis. In particular, ACM also upregulated the expression of VEGF, EGF, bFGF, and KDR in HUVECs. Therefore, these data suggest that ACM contributes to cutaneous wound regeneration.

Considering that T2D accounts for more than 90% of diabetes cases (Edlitz and Segal, 2022), a T2D skin wound injury rat model was used to assess the therapeutic effect of ACM on skin wounds. Significantly, we found that ACM could promote the skin wound healing rate. It is well-known that excessive inflammation is caused by the crosstalk of various immune cells, including neutrophils (Zhu et al., 2021), macrophages (Lv et al., 2023), and lymphocytes (Baltzis et al., 2014), which is also characterized by the high expression of various pro-inflammatory factors, including TNF-α, IL-1β, IL-6, COX-2, IL-12, and IFN-γ (Quagliariello et al., 2021; Acosta et al., 2008; Schürmann et al., 2014). In this study, we proved that these pro-inflammatory factors were highly expressed in skin wounds, which means that excessive inflammation occurred in T2D rats. More importantly, we found that ACM could reduce the excessive inflammation. In particular, the transcriptome sequencing data further confirmed that ACM-accelerated T2D skin wound healing is closely related to the downregulation of TNF and the chemokine signaling pathway. Taken together, ACM provides a new promising strategy for accelerating T2D skin wound healing, which is partly through the TNF and chemokine signaling pathway.

It was previously shown that ADSC secretome reduces scar formation in skin wound healing (An et al., 2021) by inhibiting TGF-β1 and collagen expression (Wang et al., 2024). Paracrine cytokines and extracellular vesicles (e.g., exosomes) have been reported to be the major factors in the biological effects of ADSCs on wound healing (Wang et al., 2024). In this study, we further demonstrated that ACM accelerated diabetic skin wound healing through its anti-inflammatory functions. Given that MSC-conditioned medium or secretome has a complex composition, including extracellular vesicles (containing various types of lipids, proteins, and nucleic acids) and effector molecules (e.g., PGE2 and IDO) (Kota et al., 2017; Maughon et al., 2022; Zhao et al., 2023), the enhancement of ACM in skin wound regeneration may involve multiple targets and pathways. Further studies should focus on the different components and targets of ACM in the therapeutic role in T2D skin wound repair to verify more detailed mechanisms. Recently, Yin et al. reported that ADSC exosomes promote diabetic wound healing by regulating macrophage polarization (Yin and Shen, 2024) and epidermal autophagy (Ren et al., 2024). Therefore, exosomes of ACM would play a key role in accelerating diabetic wound healing. Furthermore, before proceeding with further clinical trials or applications, it is crucial to ensure strict control over the large-scale production, stability, and quality considerations related to ACM production.

In this study, we showed that ACM could enhance vascular proliferation and angiogenesis, promote skin wound healing in type 2 diabetes, and inhibit the inflammatory response. The mechanism may involve the downregulation of the TNF and chemokine pathways.

## 5 Conclusion

In conclusion, our study demonstrates that ACM can significantly accelerate the healing of diabetic skin wounds by promoting vascular remodeling and suppressing inflammation through the TNF and chemokine pathways.

## Data Availability

The datasets presented in this study can be found in online repositories. The names of the repository/repositories and accession number(s) can be found in the article/Supplementary Material.
