# Supplementary material for: Mesenchymal stem cell-conditioned medium accelerates type 2 diabetic wound healing by targeting TNF and chemokine signaling
Source: Front Cell Dev Biol. 2025 Sep 24;13:1659444. doi: 10.3389/fcell.2025.1659444 (PMC12504491; doi:10.3389/fcell.2025.1659444)
Supplement: Supplementary file 1 [file DataSheet1.pdf]

Supporting Information

Mesenchymal stem cell-conditioned medium accelerates type 2 diabetic wound healing by targeting TNF and chemokine signaling

Long Huang[1#], Zhongbao Lin[1#], Haiyun Liu[2], Xiankun Lin[1], Naishun Liao[3*], Xiaodan Wu[4*]

[1]Department of Respiratory and Critical Care Medicine, Shengli Clinical Medical College of Fujian Fuzhou University Affiliated Provincial Hospital, Fuzhou 350001, P. R. China.

[2]Shengli Clinical Medical College of Fujian Medical University; Department of Emergency, Fujian Provincial Hospital; Fuzhou University Affiliated Provincial Hospital; Fujian Provincial Key Laboratory of Emergency Medicine, Fuzhou 350001, P. R. China.

[3]The United Innovation of Mengchao Hepatobiliary Technology Key Laboratory of Fujian Province, Mengchao Hepatobiliary Hospital of Fujian Medical University, Fuzhou 350028, P. R. China.

[4]Department of Anesthesiology, Shengli Clinical Medical College of Fujian Fuzhou University Affiliated Provincial Hospital, Fuzhou 350001, P. R. China.

[#]Contributed equally.

**Corresponding authors:* correspondence should be address to Dr. Naishun Liao, liaons046@163.com or Prof. Xiaodan Wu, wxiaodan@sina.com.

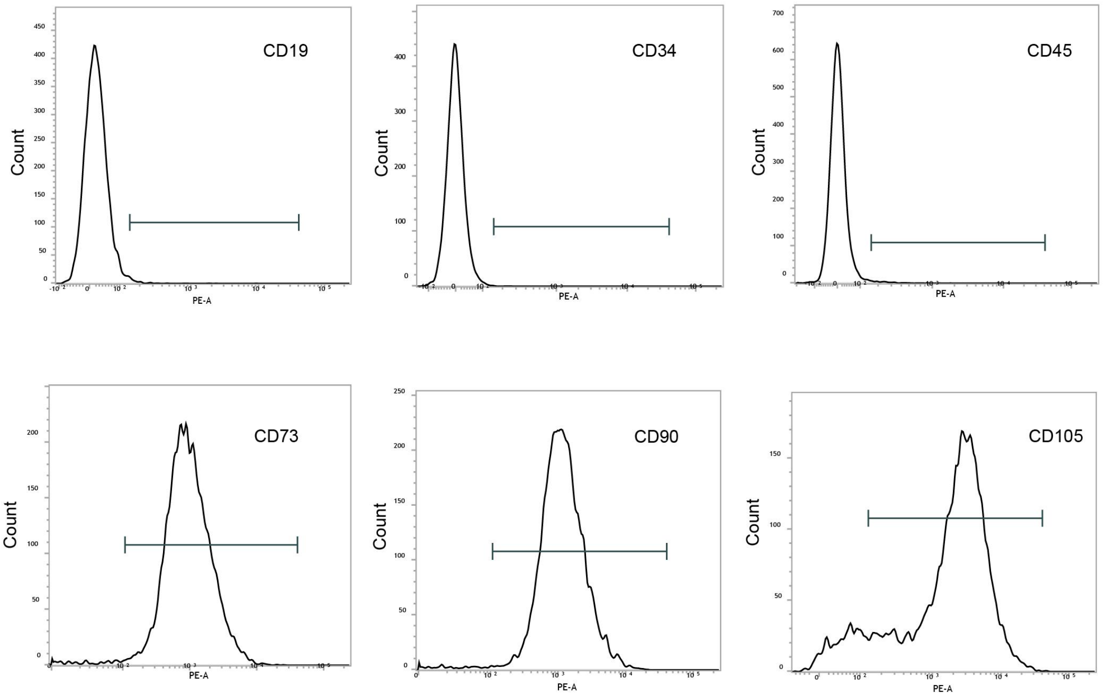

Figure S1 The characteristic of ADSCs.

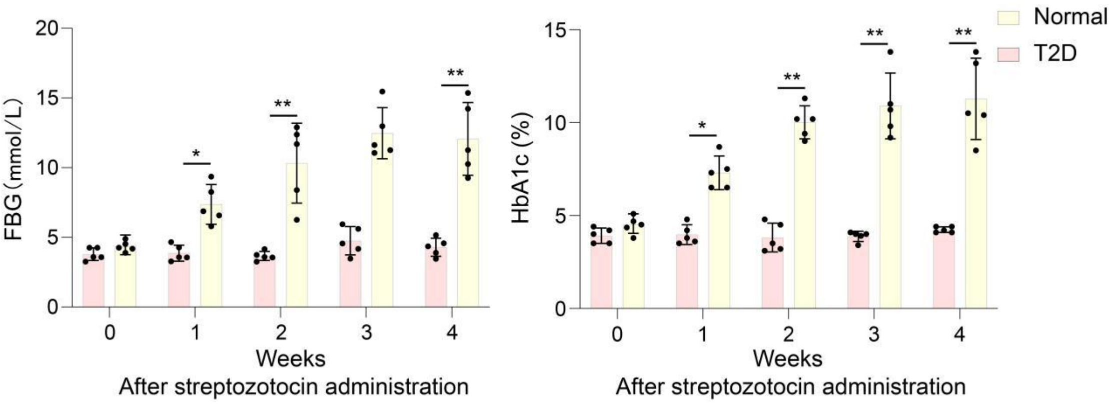

Figure S2 The FBG and HbA1c levels in T2D rats. The TBG and HbA1c levels before and after streptozotocin administration (once a week, for repeated 2 times).

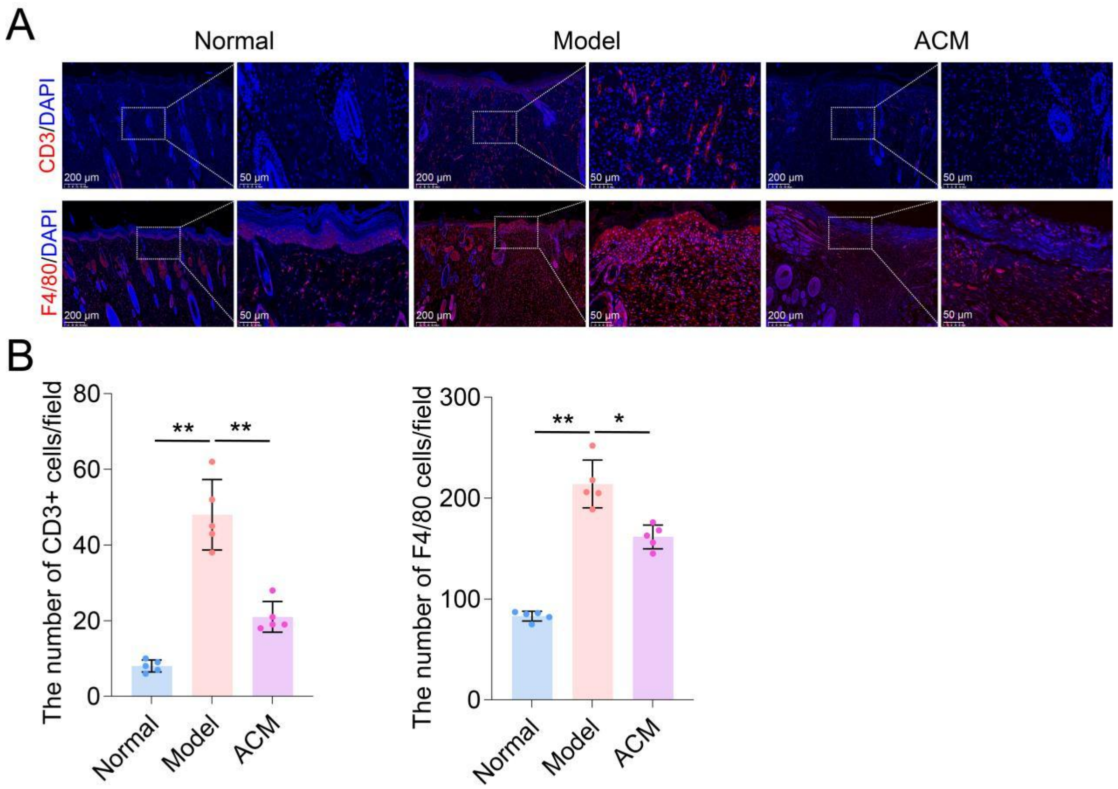

Figure S3 ACM inhibits T2D skin wound inflammation in rats. Immunofluorescence expression of $CD3^{+}$T cells and $F4/80^{+}$macrophages in skin wounds after ACM treatment. Scale bar = 200 μm and 50 μm, respectively.
